# Engineering considerations of iPSC-based personalized medicine

**DOI:** 10.1186/s40824-023-00382-x

**Published:** 2023-07-07

**Authors:** Sangbae Park, Yonghyun Gwon, Shahidul Ahmed Khan, Kyoung-Je Jang, Jangho Kim

**Affiliations:** 1 Department of Convergence Biosystems Engineering, Chonnam National University, Gwangju, 61186 Republic of Korea; 2 Department of Rural and Biosystems Engineering, Chonnam National University, Gwangju, 61186 Republic of Korea; 3 Interdisciplinary Program in IT-Bio Convergence System, Chonnam National University, Gwangju, 61186 Republic of Korea; 4 Institute of Nano-Stem Cells Therapeutics, NANOBIOSYSTEM Co, Ltd, Gwangju, 61011 Republic of Korea; 5 Department of Bio-Systems Engineering, Institute of Smart Farm, Gyeongsang National University, Jinju, 52828 Republic of Korea; 6 Institute of Agriculture & Life Science, Gyeongsang National University, Jinju, 52828 Republic of Korea

**Keywords:** Personalized medicine, Engineering strategies, Induced pluripotent stem cells, Next-generation therapeutics

## Abstract

Personalized medicine aims to provide tailored medical treatment that considers the clinical, genetic, and environmental characteristics of patients. iPSCs have attracted considerable attention in the field of personalized medicine; however, the inherent limitations of iPSCs prevent their widespread use in clinical applications. That is, it would be important to develop notable engineering strategies to overcome the current limitations of iPSCs. Such engineering approaches could lead to significant advances in iPSC-based personalized therapy by offering innovative solutions to existing challenges, from iPSC preparation to clinical applications. In this review, we summarize how engineering strategies have been used to advance iPSC-based personalized medicine by categorizing the development process into three distinctive steps: 1) the production of therapeutic iPSCs; 2) engineering of therapeutic iPSCs; and 3) clinical applications of engineered iPSCs. Specifically, we focus on engineering strategies and their implications for each step in the development of iPSC-based personalized medicine.

## Introduction

Personalized medicine, also referred to as precision medicine, offers tailored medical treatment that considers the clinical, genetic, and environmental characteristics of patients [1]. Advances in biotechnology and growing awareness of quality of life have promoted a paradigm shift from conventional medicine toward personalized medicine. Conventional medicine provides patients with guidelines established through empirical- and mechanism-based treatment [2]. Although this approach considers patient heterogeneity, it has limited potential for optimized therapy or treating specific cases [3]. Personalized medicine has significant advantages over conventional medicine by providing optimized therapy that enhances treatment safety and efficacy while reducing adverse effects. In addition, personalized medicine can be applied to ultrarare diseases as well as preventive medicine through disease modeling and diagnosis [4]. Therefore, personalized medicine improves patient health by providing customized therapies according to an individual’s biological information [5], resulting in improved recovery time and clinical failure rates [6, 7].

In 2006, Takahashi and Yamanaka first reported the generation of iPSCs by delivering four key transcription factors (OCT3/4, SOX2, c-MYC, KLF4) into murine adult fibroblasts using retroviral vectors [8]. These generated iPSCs exhibited typical embryonic stem cell-like characteristics, including the morphology, growth behavior, and expression of distinctive embryonic stem cell markers [9]. iPSCs also have the potential for self-renewal and pluripotency. The discovery of iPSCs led to dramatic improvements in personalized medicine [9]. As iPSCs are derived from a patient’s cells, they are promising candidates for disease modeling, drug screening, and genetic modification. iPSCs also have significant advantages; for example, they are unencumbered by ethical issues (unlike embryonic stem cells), they can differentiate into almost every cell type, and they are highly immunocompatible because they are harvested and reprogrammed from the patient’s own cells [10].

Despite abundant research on iPSCs in relation to personalized medicine, numerous obstacles remain to the successful production of high-quality iPSC therapeutics; these include reprogramming efficiency, expansion, differentiation capabilities, and quality control [11]. These limitations impede the development of high-quality iPSC products. However, engineering strategies have the potential to overcome these limitations and facilitate the widespread use of iPSCs in personalized medicine [12]. The development process of iPSC-based personalized medicine can be divided into three distinct steps. The first step is the production of therapeutic iPSCs. In this step, patient biopsies can either be reprogrammed into iPSCs in the hospital (in-hospital iPSCs) or sent away for the production of commercialized therapeutic iPSCs (which includes commercialized iPSCs and personalized iPSC line banking) [13, 14]. Various supporting techniques are used to produce therapeutic iPSCs, such as tissue treatment, reprogramming, expansion, and automated systems for iPSC production [15]. The second step is the engineering of therapeutic iPSCs. Engineering strategies such as the paracrine effect [16], differentiation [17], biomodulation [18], and pharmaceuticals [19] offer various opportunities for applying therapeutic iPSCs to personalized medicine. These strategies can either improve the performance of iPSC therapeutics or impart new functions. The third step is the clinical application of engineered iPSCs. Individual or combined engineering strategies can be used in clinical applications. The application of engineered iPSCs could involve personalized tissue regeneration, personalized cancer therapy, and drug development identified through drug screening of the iPSCs derived from the patient’s cells [20].

**Fig. 1:**
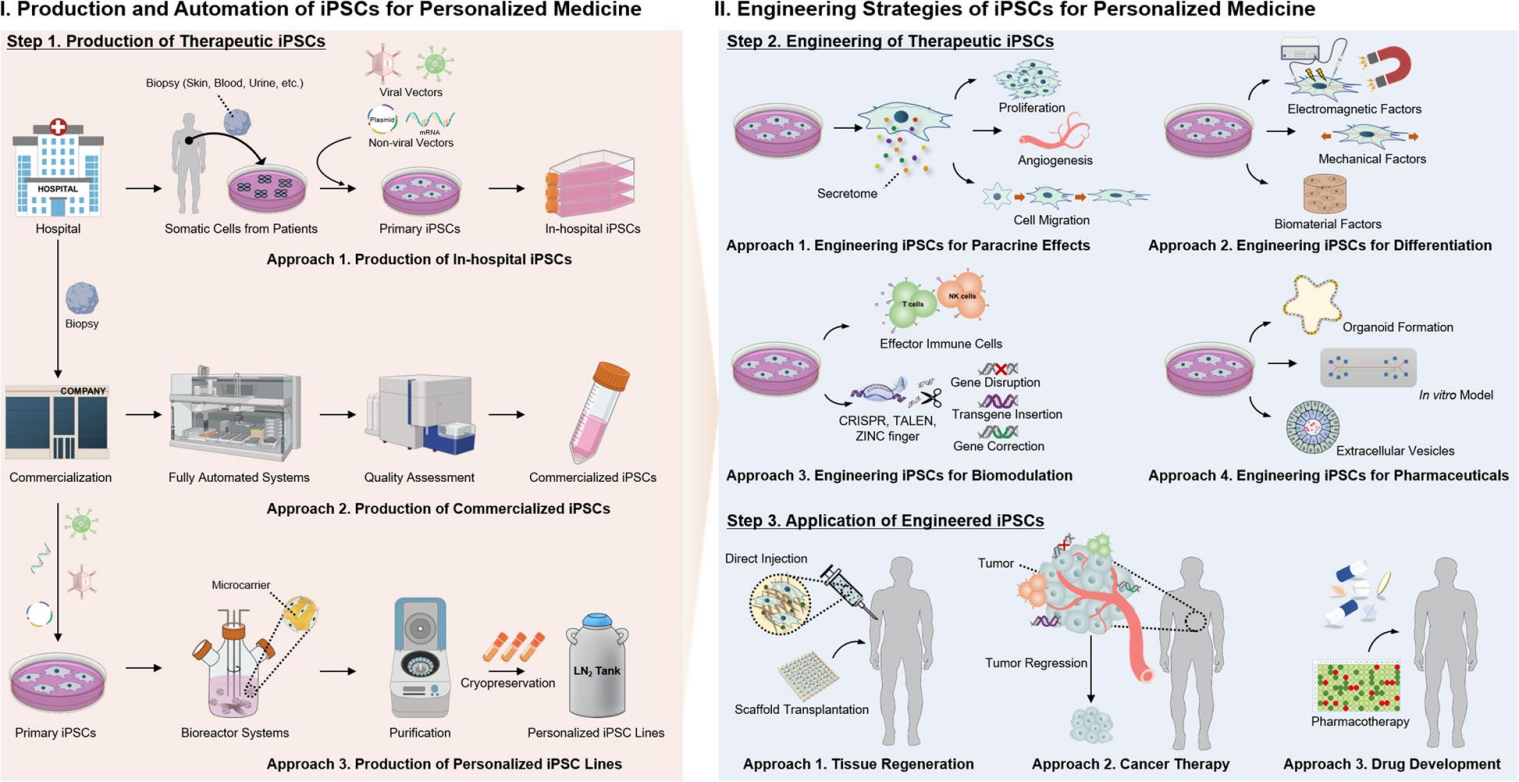
Overview of iPSC engineering steps for personalized medicine. Step 1: Production of therapeutic iPSCs (three approaches). Approach 1. Production of in-hospital iPSCs. Patient biopsy collected from skin, blood, liver, hair follicles, or urine is reprogrammed by reprogramming factors integrated with viral and non-viral vectors for the production of iPSCs. In-hospital iPSCs are then expanded for further use. Approach 2. Production of commercialized iPSCs. Patient biopsy is collected from the hospital and sent to a company for commercialization. Fully automated processes are used for commercialized iPSC production, followed by a quality assessment. Approach 3. Production of personalized iPSC lines. Patient biopsy is collected from the hospital and sent to a company. Samples are reprogrammed to produce commercialized iPSCs. The commercialized iPSCs are further expanded using bioreactor systems. Purification stages should be performed before the establishment of personalized iPSC lines. Step 2. Engineering of therapeutic iPSCs (four approaches). Approach 1. Engineering iPSCs for paracrine effects. iPSCs release different types of secretomes and regulate cell fate, such as proliferation, angiogenesis, and cell migration. Approach 2. Engineering iPSCs for differentiation. iPSCs are differentiated by electromagnetic factors, mechanical factors, and biomaterial factors. Approach 3. Engineering iPSCs for biomodulation. Different types of engineering techniques are used for biomodulation. iPSC-derived immune cells (T-cells, NK cells) are used for immunomodulation, whereas CRISPR, TALEN, and ZINC fingers are used for genetic modification, which includes disruption, transgene insertion, and gene correction. Approach 4. Engineering iPSCs for pharmaceuticals. Engineering strategies such as organoids, in vitro models, and extracellular vesicles are used for pharmaceuticals. Step 3. Application of engineered iPSCs (three approaches used in various combinations). Approach 1. Tissue regeneration. Engineered iPSCs can either be directly injected or transplanted with scaffolds. Approach 2. Cancer therapy. iPSCs are used for tumor regression through various combinations of engineering strategies. Approach 3. Drug development. Engineered iPSCs are used for drug development and drug screening

While other reviews on iPSCs have highlighted on biomaterials, generation techniques, and clinical applications, we focused on how engineering strategies can be comprehensively applied in iPSC-based personalized medicine ranging from iPSC preparation stage to clinical applications. In this review, we provide a classification to categorize the existing researches with state of the art engineering technologies. In this review, we highlight how engineering strategies have been applied to advance iPSC-based personalized medicine by categorizing the development process into three distinctive steps. For each step of iPSC-based personalized medicine development, we focus on the engineering considerations and their implications (Fig. 1). First, we introduce the preparation of therapeutic iPSCs, which include in-hospital iPSCs, commercialized iPSCs, and personalized iPSC lines, as well as supporting techniques. We then discuss recent progress in engineering strategies for generating iPSC functions suitable for personalized applications. Third, we review recent progress in the clinical application of iPSCs for personalized medicine. Finally, we discuss the remaining limitations, challenges, and prospects for engineering strategies in iPSC-based personalized medicine.

## Preparation of iPSCs for personalized medicine

iPSC preparation is regarded as the primary stage in the therapeutic application of iPSCs for personalized medicine. Therefore, in this section, we focus on “Step 1: Production of therapeutic iPSCs” (Fig. 1). There are three major approaches for producing therapeutic iPSCs: 1) production of in-hospital iPSCs; 2) production of commercialized iPSCs; and 3) production of personalized iPSC lines. Each approach involves different procedures and additional scientific processes for the preparation of therapeutic iPSCs (Table 1). A common initial stage is the collection of patient biopsies from different parts of the body, such as the skin, blood, liver, hair follicles, or urine. After biopsy collection, the following stage involves the production of either in-hospital iPSCs or commercialized therapeutic iPSCs [13, 14]. Commercialized therapeutic iPSCs can either be produced from fully automated processes (commercialized iPSCs) or established as personalized iPSC lines for personalized use. Both in-hospital and commercialized therapeutic iPSCs hold great promise for personalized medicine. The approach to produce in-hospital iPSCs could provide an immediate supply of patient-specific cells. On the other hand, commercializing the therapeutic iPSCs can significantly increase the productivity and quality compared to those of in-hospital iPSCs.

**Table 1:** Preparation of iPSCs for personalized medicine

iPSC Preparation Stage	Techniques	Features	Outcomes	References
Reprogramming Techniques	Lentiviruses	iPSCs produced from adult fibroblast	Treatment with valproic acid increased cell proliferation	[21]
	Lentiviruses	iPSCs produced from mouse tail-tip fibroblast	Porphyra 334 increased the effectiveness of cell reprogramming	[22]
	Sendai viruses	iPSCs produced from peripheral blood mononuclear cells	Heterozygous frameshift mutation in C19orf12 brought by the insertion	[23]
	Episomal plasmids	iPSCs produced from mononuclear cells	No serious adverse events related to CYP-001	[24]
	Episomal plasmids	iPSCs produced from mouse embryonic fibroblast with small molecules	Tenfold increase in reprogramming efficiency	[25]
	Episomal plasmids	iPSCs produced from a peri-infarct area	Endogenous brain repair, reduced inflammation and glial scar formation	[26]
	Episomal plasmids	iPSCs produced from an amyotrophic lateral sclerosis patient’s cell	5-hydroxymethyl cytosine levels increase the reprogramming	[27]
	Circular DNA plasmids	iPSCs produced from B16F10 cells	Did not form teratomas, suppression of tumorigenic abilities	[28]
	mRNA	iPSCs produced from neurons	Purified and differentiated into hair cell-like cells and neurons	[29]
	mRNA	iPSCs produced from urine-derived cells	Generating feeder-free bulk hiPSC lines without genomic abnormalities	[30]
	Small molecules	iPSCs produced from mouse embryonic fibroblasts	Facilitates both in vitro and in vivo alterations in cell fate	[31]
	Small molecules	iPSCs produced from neural stem cells	Melatonin promoted N-iPSC proliferation	[32]
	CRISPR-Cas9	iPSCs produced from skin biopsies	Generate gene-edited hiPSCs from carrying a point mutation	[33]
	Epigenetic modifications	iPSCs produced from mouse fibroblasts	Reconfigurations rapidly propel deterministic reprogramming toward naive pluripotency	[34]
	*C9ORF72*-mutated	iPSCs produced from fibroblasts and peripheral blood cells	iPSCs and motor neurons derived from the two tissues showed identical properties and features	[35]
	CtIP protein	iPSCs produced from mouse embryonic fibroblast	DNA repair fidelity to both human and mouse iPSCs	[36]
	hiPSC3F-FIB or hiPSC4F-FIB	iPSCs produced from human fibroblasts and fetal neural stem cells	Does not alter subsequent differentiation into neural lineages	[37]
	Integrated at the AAVS1 locus	iPSCs produced from neuron cells with neurogenin 2 transgene	In LOPAC, tau-lowering compounds has been identified	[38]
	OSKM factors, absence of LIF	iPSCs produced from mouse embryonic fibroblasts	No tumor formation but formation of clear hyaline, hypertrophic cartilage	[39]
	Six different reprogramming methods	iPSCs produced from fibroblasts and reprogramed by Lentivirus, Sendai, MiniCircle, Episomal, mRNA, and microRNA	Best results showed by Sendai-virus-based reprogramming	[40]
iPSC Expansion	Stirred based bioreactors	Expansion of macrophages generated from peripheral blood CD34 + cells-derived iPSCs	Highly pure CD45 + CD11b + CD14 + CD163 + cells, act like professional phagocytes	[41]
	Stirred based bioreactors	1 ~ 4 × 10^7^ iPSCs-derived macrophages can be harvested weekly	The ongoing, precise creation of iPSC-Mac populations	[42]
	Vertical-wheel bioreactors	Expansion of human iPSCs as aggregates in single-use bioreactors	Expand iPSCs to expand cells up to 2.3 × 10^6^ (Maximum cell density)	[43]
	Vertical-wheel bioreactors	With a cumulative cell expansion of 1.06 × tenfold in 28 days, the expansion is 30 times in 6 days	Rapid generation of high-quality hiPSCs	[44]
	Vertical-wheel bioreactors with GelMA microcarriers	8-day cell growth that increased 16-fold, differentiation, and immune modulation capacity	Robust, scalable, and cost-effective with translational potential	[45]
	Spinner flask bioreactors	Primary macrophages with cytokine release, phagocytosis, and chemotaxis	Synthesis of genetically altered, iPSC-derived macrophages on a large scale	[46]
	Hydrogel-based 3D culture	Promotes endothelial-network formation and identifies angiogenesis inhibitors	Superior sensitivity and reproducibility over Matrigel	[47]
	Hydrogel-based 3D culture	Fibroblasts formed tiny clusters, spheroids, short segments and on day 20, lengthy segments	The production of closed, inexpensive devices and iPSCs is more rapid, reliable, and scalable	[48]
	Transwell-based 3D culture	In vivo, ex vivo, and in vitro nephrogenic potential, able to produce metabolites that resemble urine	A platform for renal disorders, drug discovery, and human nephrogenesis	[49]
	Multi-culture flasks	Glycogen synthase kinase-3b suppression, CHIR99021 causes a massive proliferation of hiPSC-CMs in vitro (100- to 250-fold)	Expanding hiPSCs for use in tissue engineering and drug screening in a large-scale	[50]
	Chemically defined culture medium	Human skin fibroblasts or peripheral blood mononuclear cells are used to create iPSCs	Differentiation into three embryonic germ layers	[51]
	Chemically defined culture medium	hiPSCs with increased metabolic activity derived from blastocysts or somatic cells	GMP-friendly methods for the manufacturing and processing of therapeutic hiPSC	[52]
	Plate shaker based liquid handler	Cell seeding, splitting, expansion, differentiation image-based multiparametric screening	NPC's neuronal differentiation in 3D midbrain organoids and 2D culture	[53]
	Culture dishes coated with polymer	Create particles with zwitterionic polymer that resemble hyaline cartilaginous tissue and type II collagenopathy	Mass production of chondrocytes and cartilaginous tissues used for drug screening	[54]
Establishment of iPSC Line	Mutagenized iPSC line	CRISPR/Cas9-dependent reprogramming iPSCs	Development of loss-of-function disease models	[55]
	Heterozygous COL1A1 mutation iPSC lines	Karyotype expressed pluripotency markers	Osteogenesis imperfecta disease mechanisms	[56]
	Homozygous/heterozygous iPSC lines	CRISPR-Cas9 dependent reprogramming	Generation of two isogenic iPSC lines	[57]
	KCNA2 mutation iPSC lines	KCNA2 point mutation for produce induces pluripotent stem cells	Expression of pluripotency markers, differentiation into three germ layers	[58]
	Footprint-free iPSC lines	Whole-genome sequencing-based annotated iPSCs lines	Personal Genome Project Canada for personalized iPSC line	[59]
	cGMP-manufactured hiPSC lines	Can produce retinal cells	A human iPSC line that has been used to create transplantable photoreceptors	[60]
	CD34 + hematopoietic cells iPSC lines	CD34 + hematopoietic stem cells from peripheral blood	The production and characterization of three hiPSC lines compatible with GMP	[61]
Process Automation	Fully automated	Microcolonies throughout a 7-day period, sensitivity of 88%, and 98% detection specificity	label-free sensing and mother colony maintenance	[62]
	Fully automated	Retinal pigment epithelial cells are produced using TECAN Fluent automated cell culture	A commercially available platform called end-to-end workflow	[63]
	Automated reprogramming process	Platform for differentiated cells that uses robotics and human involvement	Population-scale personalized iPSC line	[64]
	Automated reprogramming, isolation, and expansion process	Expression of the TRA-1–60 marker for pluripotent stem cells	Commercialized iPSCs line establishment	[65]
	Automated cell culture process	The cell yields, aggregation rates, and expression were higher in non-centrifugation populations	Successfully transferred to independent laboratories	[66]
	Automated cell culture process	Differentiated into dopaminergic neurons, pancreatic cells, and pancreatic hormones	Differentiated into three germ layers	[15]
	Automated quality assessment process	A *k*-NN classifier with three potential classes has the best accuracy (62,4%) for classification	Automatic evaluation of iPSC colony image quality	[67]
	Biologically inspired AI-based automated process	More adaptable and capable of resolving a wide variety of optimization issues	A necessary simulation is introduced along with the proper model fitting technique	[68]

### General techniques for iPSC preparation

#### Reprogramming techniques

Several reprogramming techniques are available for producing iPSCs, including biochemical, chemical, and mechanical reprogramming approaches. One of the simplest procedures produces iPSCs from adult human dermal fibroblasts, where four Yamanaka transcription factors (OCT3/4, SOX2, c-MYC, KLF4) play a key role [8]. Countless studies have established reprogramming processes based on different viral (Lentivirus, Sendai) and non-viral (MiniCircle, Episomal, mRNA, and microRNA) reprogramming vectors [40]. Occasionally, reprogramming factors may diverge and some additional transcriptomic factors and/or small molecules may be added to achieve the outcome. For example, Armijo et al. suggested that patient fibroblasts can be reprogrammed using a lentivirus encoding the reprogramming factors OCT4, SOX2, c-MYC, KLF4, NANOG, and LIN28 supplemented with small molecules [69]. Recently, for a patient carrying the atrial septal defect mutation for congenital heart disease in the GATA4 gene, a urine sample was reprogrammed by lentiviral particles containing human POU5F1, SOX2, KLF4, c-MYC, and RFP to produce iPSCs via epigenetic modification [70]. Currently, the most promising and reliable viral vector for reprogramming cells to produce in-hospital iPSCs is the Sendai virus. Previous studies have demonstrated the process of sampling fibroblasts through skin punch biopsies and reprogramming them using the Sendai virus expressing the four major factors OCT4, SOX2, KLF4, and c-MYC [71–73]. In addition, a study comparing six reprogramming techniques according to their transcriptomic and epigenomic differences (lentivirus, Sendai, MiniCircle, episomal, mRNA, and microRNA) found that Sendai-virus-based reprogramming was the optimal method for generating human iPSCs [40]. Moreover, a T-cell reprogramming technique based on the Sendai virus has been employed for the generation of iPSCs, whereby a small amount of human peripheral blood was collected and reprogrammed by activated T-cells and mutant Sendai virus encoding human OCT3/4, SOX2, KLF4, and c-MYC [74]. Retroviral reprogramming is another viral-based reprogramming technique for producing iPSCs. For example, a previous study isolated dermal fibroblasts from patients carrying parkin gene mutations, then reprogrammed these cells using retroviruses carrying OCT4, SOX2, KLF4, and c-MYC, providing a potential therapy for the treatment of Parkinson’s disease [75]. Patient-specific pluripotent stem cells for neurological disease applications have been produced through several common reprogramming methods, such as retroviral and lentiviral integration of OCT4, SOX2, c-MYC, KLF-4, Cre-loxP recombination, PiggyBac transposon, small molecules, protein-based, and microRNA factors [76].

Episomal-based reprogramming is also a prominent reprogramming method for the production of iPSCs. Through this method, patient samples are collected and reprogrammed by an episomal non-integrated procedure. For example, to produce neuron cells, patient biopsies were collected from peripheral blood and reprogrammed using episomal plasmids encoding the transcription factors OCT3/4, SOX2, KLF4, LIN28, and L-MYC, resulting in their successful differentiation into neurons [77]. Moreover, to determine targeted iPSCs, a reprogramming technique combining episomal plasmids with small molecules has been established for adult fibroblasts [25]. For the clinical treatment of strokes, which represent a severe health problem in the modern world, in-hospital iPSCs can be generated from patient fibroblasts. That is, an electroporation reprogramming technique has been used for the integration of episomal plasmid vectors comprising OCT3/4, KLF4, SOX2, L-Myc, and Lin28 [26]. Esanov et al. suggested reprogramming patient fibroblasts using non-viral, integration-free episomal plasmids combined with OCT4, NANOG, TRA-1–81, and SSEA4 [27]. mRNA is another key tool for iPSC reprogramming. One study compared two different reprogramming methods for fibroblasts collected from the patient’s foreskin; one sample group reprogrammed by transduction with an integrating lentiviral vector encoding SOX2, OCT4, LIN28, and NANOG, whereas the other was reprogrammed using non-integrating mRNAs encoding SOX2, OCT4, LIN28, KLF4, and c-MYC. They found that the mRNA-reprogrammed sample was differentiated into otic cell types and concluded that it was the safest way of inducing pluripotency [29]. Another study involving the reprogramming of a urine-derived cell line concluded that the mRNA reprogramming technique is a fast and reliable method [30]. Another study produced in-hospital iPSCs from a 50-year-old female patient by reprogramming somatic fibroblasts via the transfection of synthetically modified mRNA encoding transcription factors [78].

#### Expansion techniques

**Fig. 2:**
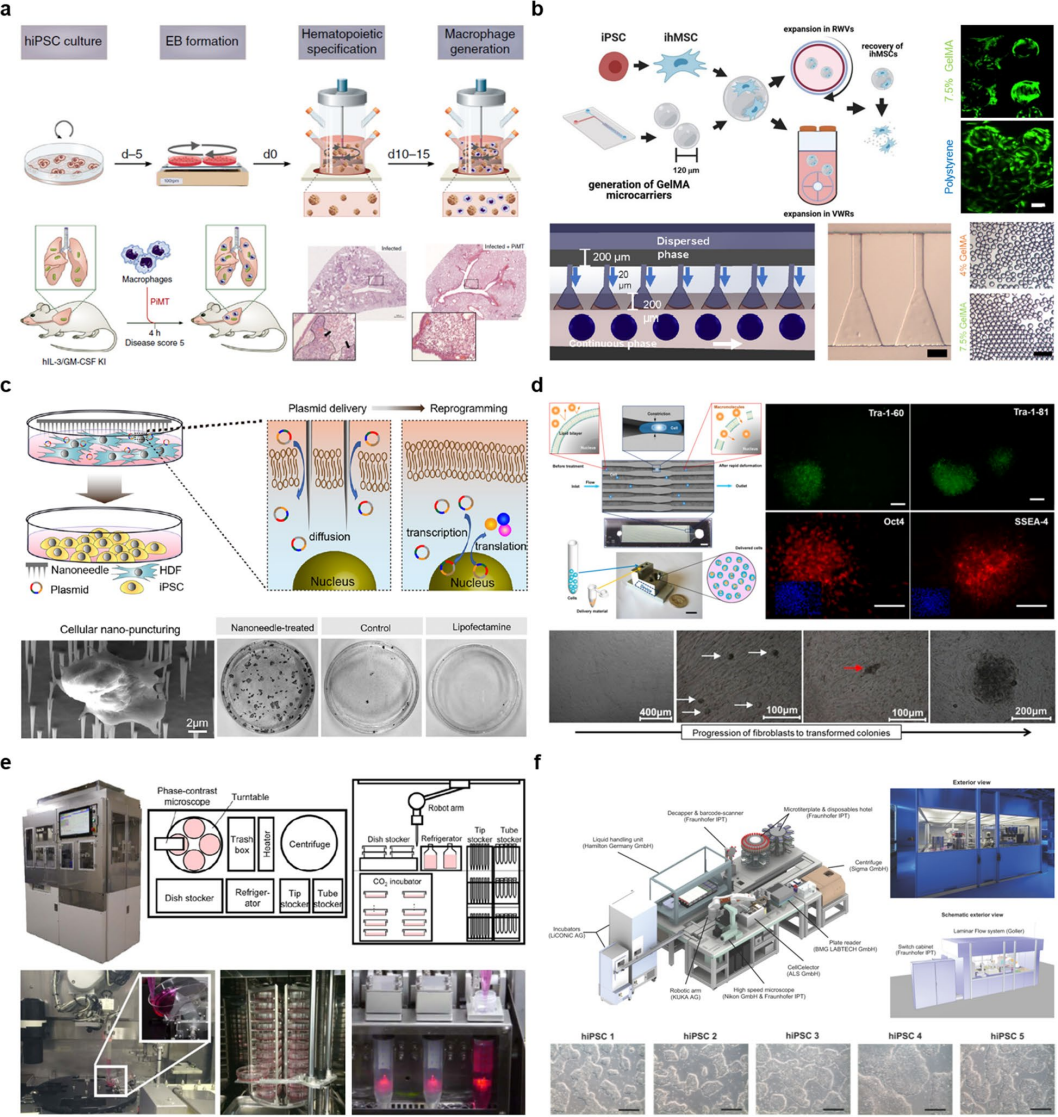
Preparation and automation of iPSCs for personalized medicine. **a** Mass production of human iPSCs in stirred-tank bioreactors. Hematopoietic differentiation of human iPSCs in stirred-tank bioreactors. Reproduced with permission from Ref. [41]. **b** Scalable system for iPSC generation, where uniform gelatin methacryloyl microcarriers are fabricated via the microfluidic-based emulsification process. Reproduced with permission from Ref. [45]. **c** Nanopuncture-assisted iPSC reprogramming via the intracellular delivery of mini-intronic plasmids (MIP) and human neonatal dermal fibroblast (HDF) cells. Reproduced with permission from Ref. [79]. **d** A microfluidic device is designed to create a transient membrane hole in the cell surface when they are passed through the device because of the rapid deformation of cells. Reproduced with permission from Ref. [80]. **e** Long-term maintenance of human iPSCs by an automated cell culture system, which helps human iPSCs maintain their undifferentiated state for 60 days. Human iPSCs generated by this system can differentiate into three germ layer cells as well as dopaminergic neurons and pancreatic cells. Reproduced with permission from Ref. [15]. **f** An automated procedure for the iPSC line expansion through culturing and reprograming from human fibroblast in a controlled clean room environment. This platform is designed with a liquid handling unit, incubator, robotic arm, microscope, picker, plate reader, centrifuge, and microtiter plate. Reproduced with permission from Ref. [65]

Continuous production of iPSCs or iPSC-based products is another important consideration for increasing cost-effectiveness of iPSCs. From this point of view, the expansion of iPSCs is recognized as a significant factor in the field of personalized medicine for continuous treatment. The best way to solve this issue is to use an expansion system that has been used for cell expansion, optimized for iPSCs. For example, human iPSCs can be expanded to 2.3 × 10^6^ (maximum cell density) using vertical-wheel bioreactors within 1 to 7 days and used for continuous innovative cell-based treatment [43]. Similarly, iPSC-derived macrophages can be produced using stirred-tank bioreactors within 10 to 15 days and used for continuous innovative cell-based treatment (Fig. 2A) [41]. Microcarrier-based platforms are also popular for iPSC production. For example, degradable gelatin methacryloyl microcarriers were employed in a reliable, scalable, and affordable method for the expansion and rapid harvest of iPSCs using an inexpensive and bespoke microfluidic step-emulsification apparatus, which achieved expansion of 8.8 to 16.3 times within eight days (Fig. 2B) [45]. In any commercialized product, a high yield within a short time is desirable. Therefore, researchers have developed an expansion-based spinner culture medium approach for the high-yield, large-scale generation of macrophages using iPSCs. These macrophages exhibit cytokine release, phagocytosis, and chemotaxis for drug screening [46]. Moreover, the inflection of signaling pathways through different enzymatic or gene editing activities is another reliable technique for the production of iPSCs. In addition, modulating signaling pathways by inhibiting glycogen synthase kinase-3b with CHIR99021 can promote human iPSC neural progenitor proliferation in a cell density-dependent manner, enhancing iPSC expansion by 10 to 25 times, which is beneficial for extensive drug screening and tissue engineering activities [50].

### Production of in-hospital iPSCs

The main purpose of in-hospital iPSCs is an immediate use of patient-derived iPSCs to promote therapeutic effect. In this approach, appropriate biopsy location, reprogramming technique, and additional factors to increase reprogramming efficiency should be considered for the successful production of in-hospital iPSCs. Biopsies collected from different parts of the body are considered somatic cells. These primary somatic cells can be reprogrammed into iPSCs by different types of reprogramming factors with the help of viral and non-viral vectors [40]. In-hospital iPSCs may also be subjected to additional expansion processes using tissue culture dish or multi-culture flasks for their immediate use in the hospital [14]. The biopsy location is an important consideration when producing in-hospital iPSCs. For targeted iPSC production, Sharma et al. demonstrated that, in patients with TRNT1-associated Retinitis pigmentosa, skin samples should be collected from the upper, non-sun exposed arm and reprogrammed by viral transduction with the transcription factors OCT4, SOX2, KLF4, and c-MYC [81]. Human peripheral blood is considered a major source of sample collection because it involves the most accessible and least invasive procedure for in-hospital iPSC production by a non-integrating episomal plasmid approach with SOX2, KLF4, L-MYC, LIN28, and EGFP reprogramming factors [82]. Several researchers have proposed novel strategies to increase reprogramming efficiency. For example, Wang et al*.* demonstrated a nanoscale puncturing strategy for the efficient production of in-hospital iPSCs (Fig. 2C). Specifically, after collecting patient fibroblast cells, an integration-free plasmid containing OKSM reprogramming factors along with hairpin RNA p53 was used for cellular reprogramming, and diamond nanoneedles were used for cell puncturing [79]. Recently, various reprogramming tools have been suggested to boost iPSC generation technology. With the help of bioinformatics tools, GBX2, NANOGP8, SP8, PEG3, and ZIC1 were used for iPSC generation in a patient with Parkinson’s disease, resulting in a remarkable increase in the number of iPSC colonies [83]. In addition, a microfluidic approach has been developed by which cells can pass through the channel of 30 to 80% smaller diameter and creates transient holes to defuse materials into the cytosol. Through this approach, reprogramming efficiency has increased 10-folds compared to that of electroporation (Fig. 2D); this technique enables the reliable and affordable production of iPSCs for without using vectors [80]. In summary, for the successful production of in-hospital iPSCs, it is important to consider the biopsy location, reprogramming technique, and additional transcriptomic factors.

### Production of commercialized iPSCs

The main purpose of commercialized iPSCs is to increase the productivity and quality of iPSCs through commercialization. In this approach, appropriate expansion techniques, automated production processes, and quality control should be considered for the successful production of commercialized iPSCs. Briefly, biopsies collected from hospital are sent to a company for commercialization. Various fully automated processes (e.g., reprogramming, proliferation, and expansion) are employed to produce commercialized iPSCs [62]. After the fully automated processes, quality assessment is a major requirement for the successful commercialization of iPSCs and for ensuring patient safety [67]. Many scientists have developed fully automated or semi-automated iPSC production lines [64]. Compared to manual systems, fully automated systems have many advantages for reducing contamination. Indeed, a completely automated technology has been developed that allows human iPSCs to remain undifferentiated for 60 days under an automated culture system (Fig. 2E) [15]. Humans are a major source of biological contamination; therefore, delaying human involvement at the biomaterial production site is highly beneficial. To achieve this aim, a fully automated system has been designed for the generation of footprint-free hiPSCs, ranging from human fibroblasts expansion, isolation, and reprogramming. A high-speed microscope and image-based dilution calculation confirmed the in-processes quality control. Through this process, iPSCs expressed sustainable pluripotency for at least 5 weeks. (Fig. 2F) [65]. In addition, artificial intelligence (AI)-based machine learning techniques are useful for the fully automated production of commercialized iPSCs, with a *k*-NN classifier achieving a classification accuracy of 62.4% [67]. Moreover, Truong et al. presented a repeatable and scalable procedure for performing human iPSC culture and differentiation using TECAN Fluent automated cell culture workstations. This technique generated patient-derived retinal pigment epithelial cells for use in drug testing and other clinical applications [63].

Quality assurance during commercialized iPSC production is vital for ensuring patient safety. For example, Elanzew et al. established a fully automated system that encompasses fibroblast expansion to in-process quality control, as well as the determination of dilution ratios. This system was subsequently used for high-quality and industrial-scale drug screening and disease modeling [65]. Currently, new technologies are commonly integrated with AI. Recently, an automated system integrated with AI-based microscopy was established for cell sorting, which boasts 88% sensitivity and 98% specificity for human iPSC identification and has widespread applications in tissue engineering, therapeutic applications, and disease models [62]. Reducing production costs is another important aspect of commercialized iPSC production. According to previous research, the use of a synthetic culture system without growth factors together with three chemicals, fewer recombinant proteins, and commercially available media can reduce costs associated with the production of commercialized iPSCs from either human dermal fibroblasts or peripheral blood mononuclear cells [51].

### Production of personalized iPSC lines

The main aim of the personalized iPSC lines is to establish an individual biobank for preventive purposes. The personalized iPSC lines can be utilized for disease models or expanded and stored in sufficient quantities for future treatment. In this approach, biopsies sent to a company undergo reprogramming, expansion, and purification to establish a patient-specific iPSC line. Numerous automated processes have been established for the required reprogramming, bioreactor-based expansion, GMP compliance quality control, and purification of iPSCs [60]. Personalized medicine uses biological data from genetic information or biomarkers according to the profile of the specific person requiring treatment or medication, enabling faster clinical decision-making. To achieve this goal, patient-specific personalized iPSC line banking is required, with some countries already establishing such iPSC lines. For example, Genetic disorder is a major cause of organ dysfunction. Therefore, a previous study created a patient-specific iPSC line by silencing mutant collagen genes related to *Osteogenesis imperfecta* through gene targeting by an adeno-associated virus [84]. Furthermore, CRISPR/Cas9-dependent insertion/deletion techniques have been used to establish personalized iPSC lines through passage-matched isogenic controls in a single step, providing a platform for the rapid development of loss-of-function disease models [55]. As part of a personal genome project in Canada, footprint-free personalized iPSC lines were established from four volunteers, which can be used to identify variant-preferred healthy control lines and specific disease settings [59]. Moreover, personalized iPSC lines can be a solution to the problem of establishing a commercial cord blood bank with no risk to the donor, thereby enabling the treatment of neonates with genetic disorders or congenital deformities [85]. Another recent study established a personalized iPSC line of therapeutic candidates for type II collagenopathy treatment. Specifically, iPSCs derived from limb-bud-like mesenchymal cells were used to produce chondrocytes and cartilaginous tissues for drug screening and tissue engineering [54].

One study generated clinical-grade personalized iPSC lines from patient-specific fibroblasts to produce iPSC-derived retinal cells within an FDA-registered, cGMP-compatible facility with xeno-free reagent in an ISO class 5 environment [86]. Moreover, Zhu et al. suggested a procedure for generating human iPSC lines from CD34 cord blood cells and differentiating them into retinal cells using small molecule-based retinal induction under cGMP-compliant conditions, thereby generating transplantable photoreceptors [60]. Another study successfully produced personalized iPSC lines under GMP-compliant conditions through the Sendai virus-based reprogramming of peripheral blood cells and their differentiation into CD34 + hematopoietic stem cells [61]. Recently, a standard protocol was developed for the production and quality control of clinical‐grade iPSC lines within a regulatory framework [87]. A quality approach to manufacturing is mandated by GMP laws, allowing businesses to reduce or completely avoid instances of contamination, confusion, and mistakes. Furthermore, the effectiveness of a GMP-compliant method of producing iPSC lines was confirmed through a phase 1 open-label clinical trial in subjects with steroid-resistant acute graft versus host disease, which represents a milestone in the production of personalized iPSC lines [24]. Generally, treating patient-specific diseases is facilitated by storing all of the genetic and immunological data of an individual. Thus, personalized iPSC line banking is the best option for personalized medicine. The major requirements for personalized iPSC line banking are as follows: a fully automated mechanism, low production costs, high affordability, high production rate in a short time, GMP compliance with no contamination, final product and in-process quality control and assessment, patient safety, and a controlled transportation and storage system.

## Engineering strategies of iPSCs for personalized medicine

In this section, we focus on “Step 2: Engineering of therapeutic iPSCs” (Fig. 1). This stage is the most important in terms of improving the function of iPSCs for personalized medicine. Here, we cover the four engineering approaches for developing optimized iPSCs according to different goals: 1) engineering iPSCs for paracrine effects; 2) engineering iPSCs for differentiation; 3) engineering iPSCs for biomodulation; 4) engineering iPSCs for pharmaceuticals. Specifically, we discuss the recent research trends and future perspectives. Approach 1 is related to the regulation of cellular behavior and function (e.g., proliferation, migration, and growth factor expression) through the paracrine effect of engineered iPSCs. Approach 2 is related to engineering techniques (e.g., biochemical, electromechanical, and biomaterials factors) for promoting the differentiation of iPSCs. Approach 3 is related to the biomodulation of iPSCs (e.g., T-cell, CAR-T-cell, NK cell, and gene modulation) for cancer therapy. Approach 4 is related to in vitro engineering tools (e.g., organoids, organ-on-a-chip models, extracellular vesicles**)** for pharmaceutical development.

### Engineering iPSCs for paracrine effects

**Fig. 3:**
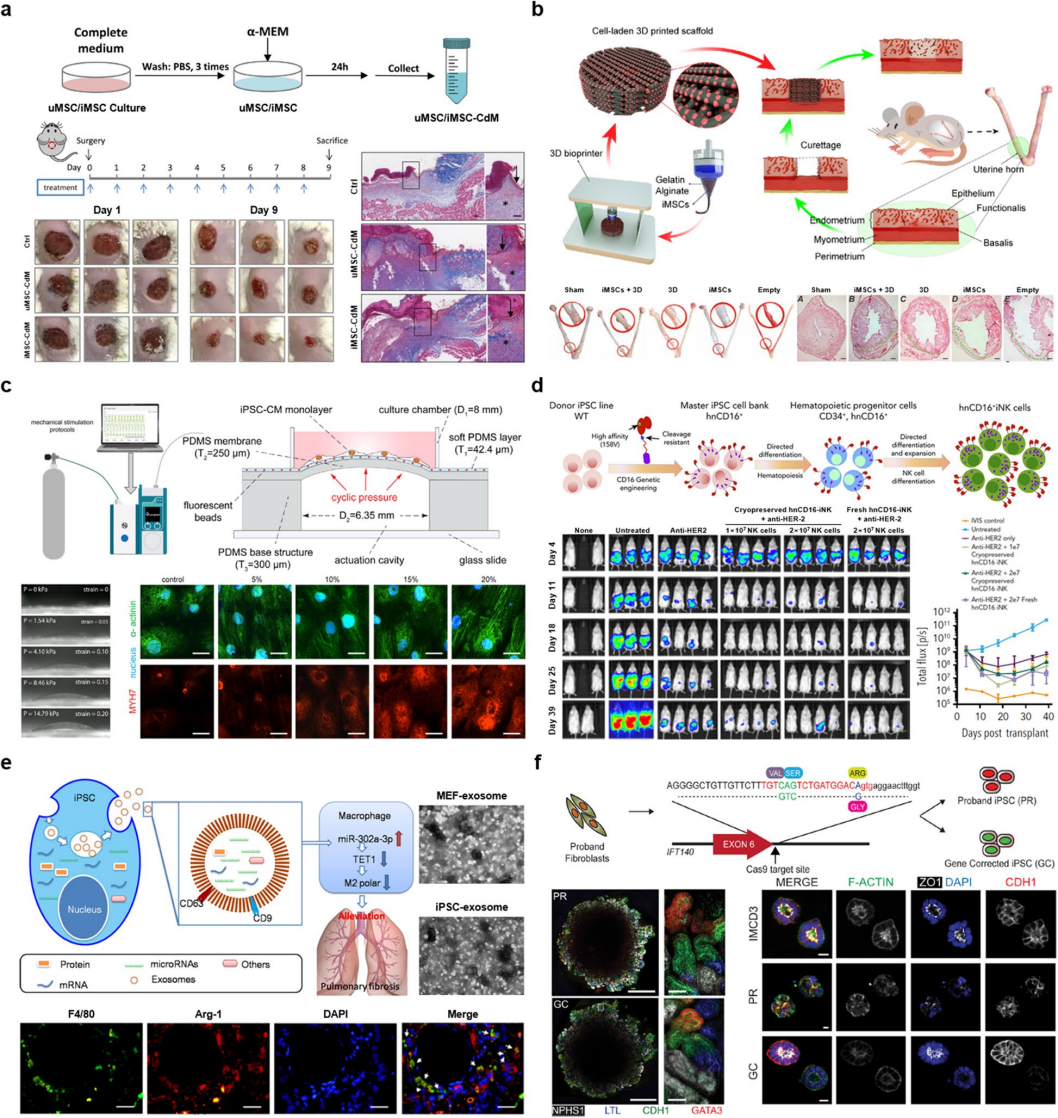
Engineering strategies and applications of iPSCs for personalized medicine. **a** Conditioned medium generated from umbilical cord-derived mesenchymal stem cells (uMSC-CdM) and iPSC-derived mesenchymal stem cells (iMSC-CdM) effectively promoted cutaneous wound healing. Reproduced with permission from Ref. [16]. **b** 3D bioprinting of a human iPSC-derived MSC-loaded scaffold for regeneration of the uterine endometrium. The preparation of human iMSC-loaded hydrogels was followed by the construction of the engineered scaffold through the 3D printing process. The engineered scaffolds were cultured in vitro for three days and then transplanted, and the structure and function of the endometrium were assessed after the repair of the uterine horn. Reproduced with permission from Ref. [94]. **c** A microdevice platform for characterizing the effect of mechanical strain magnitudes on the maturation of iPSC-cardiomyocytes. Reproduced with permission from Ref. [95]. **d** Human iPSC-derived NK (hnCD16iNK) cells produced from donor iPSC line with genetic engineering. The hnCD16iNK cells showed better antitumor activity on in vivo ovarian cancer model. Reproduced with permission from Ref. [96]. **e** Exosomes derived from iPSCs mitigate pulmonary fibrosis induced by bleomycin, with less collagen deposition. Reproduced with permission from Ref. [97]. **f** Gene correction, transcript analysis, and differentiation to kidney organoids. Patient-iPSC-derived kidney organoids show functional validation of a ciliopathic renal phenotype and reveal underlying pathogenetic mechanisms. Reproduced with permission from Ref. [98]

The main purpose of engineering iPSCs for enhancing paracrine effect is an engineering approach that can maximize the secretion of growth factors and cytokines to promote tissue regeneration. In other words, engineering iPSCs can be an effective modulator of paracrine effect. In this section, we review the engineering approaches of iPSCs to maximize the paracrine effect for personalized medicine. From this point of view, engineering iPSCs is defined as an engineering tool to maximize paracrine effects. Autologous cell therapies are arguably one of the most personalized forms of medicine, whereby a patient’s cells are used to generate a patient-specific product that is only administered back to the original donor [88]. In recent decades, iPSC-based autologous cell therapies have received substantial attention in terms of enabling patient-specific treatment for personalized medicine applications [89]. Injected iPSCs not only have therapeutic efficacy on their own but also promote the functional improvement of the surrounding environment through paracrine effect [90]. Recent studies have reported the promotion of cell proliferation, cell migration, and growth factor expression through paracrine effect of engineered iPSCs [16, 91]. Ai et al. suggested an interesting approach to maximize paracrine effect of iPSC-derived cardiomyocytes, thereby overcoming poor cell viability and engraftment rates of cell-based therapies. Prior to transplantation, they transfected VEGF mRNA to iPSC-derived cardiomyocytes to achieve overexpression of VEGF. The over-expression of VEGF facilitated cell proliferation in transplanted site, thereby promoting ventricular remodeling [92]. Also, Munarin et al. introduced a strategy of enhancing paracrine effect of implanted muscle tissue by locally delivering angiogenic factors. They found that local administration of angiogenic factors resulted in increased volumetric network density with enhanced host vascularization into implanted cardiac tissue [93]. Liang et al. also reported that the conditioned medium of reprogrammed iPSCs (CM-iPSCs) accelerates wound healing in a mouse cutaneous wound model through enhanced angiogenesis and cell migration (Fig. 3A). This study reported that growth factors in the conditioned medium of iPSCs promote skin regeneration by maximizing paracrine effect, thereby confirming tissue regeneration through paracrine effect of reprogrammed iPSCs [16].

### Engineering iPSCs for differentiation

The main aim of engineering iPSCs for differentiation is to maximize cell function, which is a key factor in regenerative medicine [99]. In this section, we review engineering approaches of iPSCs to maximize the differentiation for personalized medicine. Another form of iPSC-based personalized tissue regeneration involves the transplantation of scaffolds conjugated with iPSCs. The most significant factor to be considered in scaffold engineering is the improvement of the differentiation efficiency of iPSCs. Following the increased interest in personalized medicine, studies have investigated different engineering approaches for promoting the differentiation of iPSCs using scaffold-based biomaterials, electromagnetic, and mechanical stimulation [94, 95]. In particular, a biomaterial-based strategy for effective iPSCs differentiation requires the following conditions: excellent biocompatibility, adequate mechanical properties, good physical and chemical properties, high wear resistance, high corrosion resistance and low immune reactivity. The biomaterial-based engineering strategies under these conditions will be the most important future factor in providing efficient differentiation and safety of iPSCs for application of personalized medicine. For example, Ji et al. reported that a 3D bioprinting scaffold composed of alginate and gelatin bio-inks promoted the differentiation of iPSCs into endometrial cells (Fig. 3B). They also determined the optimal conditions for promoting iPSC differentiation and suggested the application of this natural polymer-based 3D scaffold for the repair of the uterine endometrium [94]. This study showed that natural polymer-derived bioink and bioprinting engineering technology could promote the differentiation of iPSCs, showing bioprinting technology is promising in terms of using various microstructures or biomaterials to promote iPSCs differentiation. Electrical stimulation is a powerful strategy that can be used to promote differentiation of iPSCs. They affect the voltage-gated ion channels on the cell membrane, and thus promote cell metabolism. The electrical stimulation has been reported to enhance neurogenic, cardiomyocyte, and myogenic differentiation [100–102]. Recently, several studies have introduced the effect of magnetic stimulation on neurogenic differentiation of iPSCs. Liu et al. have reported on the effect of magnetic stimulation frequency on the neuronal differentiation of iPSCs. Their study revealed that high frequency magnetic stimuli promote glutamatergic neuron differentiation, whereas low frequency and intermittent theta-burst magnetic stimuli may promote the generation of mature neuron formation [103]. Mechanical stimulation also provides significant cue that affects iPSC differentiation. For example, Dou et al. proposed a microdevice platform for characterizing the effect of mechanical strain on the cardiomyocyte differentiation of iPSCs (Fig. 3C). By applying cyclical strains of varying magnitudes (5%, 10%, 15%, and 20%) to a monolayer of iPSC-cardiomyocytes, they measured the contractile stress during mechanical stimulation and quantified the effect of different mechanical strain magnitudes on the contractility and maturation of iPSC-cardiomyocytes. Their study confirmed the correlation between mechanical strain and iPSC-cardiomyocyte differentiation through the engineering platform [95]. To summarize, previous studies have used elaborately and precisely designed engineering strategies to promote the differentiation of iPSCs and elucidate the differentiation mechanism, thereby improving the potential for the transplantation of scaffolds conjugated with iPSCs.

### Engineering iPSCs for biomodulation

The main aim of engineering iPSCs for biomodulation is to improve the function of immune substances to increase the efficiency of cell-based immunotherapy. In this section, we review engineering techniques to maximize and modulate the function of iPSCs-derived cells for cancer treatment. Cell-based immunotherapy, such as CAR T-cell therapy, has received tremendous attention in the field of cancer therapy, especially in patients who are refractory to other therapies [104]. Despite rapid advances in autologous therapies for cancer, several challenges remain, including the high cost, challenges to large-scale manufacturing, and unsuitability for lymphopenia patients [104]. iPSCs may be able to overcome these challenges because of their unique self-renewal properties and capacity to be genetically engineered [105]. Also, iPSCs can be differentiated into different immune cells, such as T-cells, NK cells, invariant NK T-cells, and macrophages [106]. Recently, engineering biomodulation studies have utilized the advantages of iPSCs for cancer therapy [18, 107, 108]. For example, human iPSC-derived NK (hnCD16iNK) cells and anti-CD20mAb improve regression of B-cell lymphoma and hnCD16iNK cells together with anti-HER2 mAb increase the survival of cancer xenograft model (Fig. 3D). From these significant findings expressed hnCD16iNK in combination with mAbs shows high effectiveness against hematologic malignancies and solid tumors [96]. Li et al. also reported that NK cells derived from human iPSCs have a typical NK cell phenotype and improved antitumor activity compared with non-CAR-expressing cells. Moreover, NK cells derived from human iPSCs significantly inhibited tumor growth, prolonged survival in vitro, and demonstrated in vivo activity similar to that of T-CAR-expressing T-cells. These studies suggest the substantial potential for NK cells differentiated from iPSCs in cancer therapy applications [18]. Furthermore, Kawamoto et al. proposed advanced methods in which cytotoxic cells are mass-produced by engineering iPSCs for the regeneration of T-cells. Specifically, iPSCs produced from T-cells inherit rearranged T-cell receptor genes; thus, all regenerated T-cells should express the same T-cell receptors with no cytotoxicity [108].

### Engineering iPSCs for pharmaceuticals

#### Drug development

Engineering iPSCs can contribute to develop innovative therapeutics with enhanced efficacy. In this sub-section, we review engineering techniques for the development of personalized pharmaceuticals or nanomedicines (e.g., cell-based therapeutics and cell-free therapeutics). Recently, iPSCs have become attractive candidates for cell therapy-based regenerative medicine. Ma et al., introduced a novel strategy of using iPSC-derived organoids for localized scleroderma therapy. According to their findings, the iPSC-derived organoids could not only alleviated skin fibrosis but also facilitated the recovery of skin-associated functions [109]. Several studies have highlighted the potential of iPSCs for developing nanomedicines. Zhou et al. proposed using iPSC-based exosomes as a latent tool for the treatment of pulmonary fibrosis (Fig. 3E); these exosomes were shown to increase the miR-302a-3p level and silence TET1 and miR-302a-3p activity, which then helps to express the iPSC-based exosomes and mitigate pulmonary fibrosis [97]. This study shows that iPSC-derived exosomes can enhance cell migration and can be a candidate for new drug development. In addition, Tang et al. proposed novel thermosensitive chitosan hydrogels loaded with iPSC-derived exosomes which can provide sustained release of miRNA present in the exosomes. The proposed hydrogels could significantly promote corneal epithelium and stroma regeneration [110].

#### Drug screening

Engineering iPSCs can also facilitate drug development by providing personalized drug screening platforms. In this sub-section, we review engineering techniques for the developing in vitro platforms (e.g., organoids, simple in vitro models, and organ-on-chips). Patient-derived iPSCs can be applied in multiple critical in vitro studies, such as in vitro disease modeling, toxicity screens, drug development, drug delivery. Furthermore, patient-derived iPSC models are more suitable for phenotypic-based drug discovery because they share the same genetic background with patients and may exhibit the same disease phenotypes. Therefore, large amounts of research have recently been conducted on iPSC-based in vitro models (e.g., organs-on-chips, organoids) for drug screening. For example, Park et al. reported the use of iPSC-based microvascular endothelium interfaced with astrocytes and pericytes in a microfluidic human-like organ-on-a-chip. Microvascular endothelium expression created the strictness by the differentiation of iPSC under hypoxic conditions. This type of chip model can be used to introduce drugs and antibiotics through the blood–brain barrier [111]. Moreover, Thomas et al. suggested a precise gene editing procedure to model renal disease based on kidney organoids differentiated from iPSCs that can validate ciliopathic renal phenotypes and reveal the underlying pathogenic mechanisms. Their kidney organoids hold great promise in high-throughput personalized therapeutic screening (Fig. 3F) [98].

## Applications of engineered iPSCs for personalized medicine

Patient-specific iPSCs can be used for the regeneration of damaged tissues [112], disease treatment [113], drug screening [114], and drug development [115], and provide solutions to overcome the limitations of conventional off-the-shelf therapy. Recent advances in biotechnology offer a variety of engineering strategies that can be used to impart or promote the function of iPSC-based products (Table 2). In this section, we focus on “Step 3: Application of engineered iPSCs” (Fig. 1). Specifically, we introduce the clinical applications of engineered iPSCs to personalized medicine, which can be classified into three approaches: 1) tissue regeneration; 2) cancer therapy; and 3) drug development. Various combinations of the engineering approaches presented in Sect. 3 can be applied to advance iPSC-based personalized medicine. For example, engineering biomaterials can create biochemically and structurally relevant microenvironments suitable for personalized tissue regeneration [116]. These engineered biomaterials can also be used as carriers to promote the survival and proliferation of transplanted cells, resulting in clinically successful outcomes [117]. In addition, genetically modified iPSCs can be used to create personal in vitro models for drug screening [118] or generate stable immune effectors for cancer therapy [119].

**Table 2:** Application of engineered iPSCs for personalized medicine

Applications	Engineering Strategy	Cell Types	Targets	Features	Outcomes	References
Tissue Regeneration	Paracrine Effects	Cardiac Cells	Cardiac tissue	Increased angiogenic potential, secretion proangiogenic and proinflammatory cytokines	Recovery from human acute myocardial infarction	[120]
		Cardiac Cells	Cardiac tissue	Up to 80% of cardiomyocyte differentiation was increased by Wnt treatment	Cardiomyocyte differentiation through paracrine factors	[121]
		Cardiac Cells	Cardiac tissue	Enhancement of promigratory, proangiogenic, and antiapoptotic	Effective recovery of damaged myocardium	[112]
		Cardiac Cells	Cardiac tissue	Secretome suppressed apoptotic cardiomyocytes > 70% locally	Heart muscle extracellular signals for cell-free treatment	[122]
		Neurons and glial cells	Neuron tissue	The Ang 1–7/Mas receptor inhibited aging and decreased neurodegenerative susceptibility	Therapeutic strategies for Parkinson’s disease	[123]
		Neurons and glial cells	Neuron tissue	Iinduced neurotrophic and neuroprotective effects and decreased the number of necrotic and apoptotic cells	encourage the development and expansion of neurites	[124]
		Neurons cells	Neuron tissue	Resource for transcriptomics on corticogenesis in 5 situations	Neurons were variable, and more developed	[125]
		Renal cells	Kidney tissue	Microbioreactor array–based multicellular differentiation	Identification of renal cells	[126]
		Murine bone cells	Bone tissue	Expression of the osteogenic genes via paracrine mechanisms	BMP-2, BMP-4, and BMP-6 gene expression is increased	[91]
		Hepatic cells	Liver tissue	The Transwell system of HE-iPSCs was separately co-cultured with MSCs and/or HUVECs	Regulate the differentiation of human hepatocytes	[127]
	Differentiation	Vascular smooth muscle cell	Vascular grafts tissue	Incorporating biodegradable scaffolds, progressive pulsatile stretching	Non-immunogenic, cellularized vascular grafts	[128]
		Vascular smooth muscle cell	Vascular tissue	PGA scaffolds express mature VSMC marker	Formation of autologous human vascular tissues	[129]
		Cardiac Cells	Cardiac tissue	Human endothelial cell patches and cell-free patches	Electrical coupling improved left ventricular function by 31%	[130]
		Cardiac and endothelial cells	Cardiac tissue	A cardiac muscle patch was created by 3D printing a scaffold with seeding cardiomyocytes and smooth muscle cells	Cell engraftment was 24.5% at week 1 and 11.2% at week 4 than cell-free scaffolds	[131]
		Lymphoblastoid cells	Cardiac tissue	Modifiable DNA methylation, chromatin accessibility, and gene expression levels	Identify the impact of chromatin accessibility specific to different cell types	[132]
		Mesodermal cells	Muscle tissue	Dystrophic mice's hearts and skeletal muscles can successfully engraft with human MiPSC	Cocktails of miRNA encourage myogenesis	[133]
		Endothelial cells	Lung tissue	Enhancing endothelial colony forming cells-built lung scaffolds with 8CPT-2Me-cAMP	Improved endothelial functionality	[134]
		hiPSC lines	Endoderm	Endoderm differentiation using a single-cell RNA-based population	Used for genetic background variability assessment	[135]
		iPSC-derived MSCs	Bone tissue	Better osseous consolidation was seen with HFF-iMSC + CPG transplantation compared with CPG alone	Express of osteopontin and bone morphogenic proteins	[136]
		Endothelial cells	Endothelial tissue	Medium supplemented VEGF is differentiated into endothelial cells	Functional cues to promote cell attachment, survival, and differentiation	[137]
		hiPSCs	Inner ear hair cells	Using CRISPR/Cas9, the MYO15A mutation was genetically fixed, saving the morphology and function	Gene mutation-based deafness can be functionally restored	[138]
	Biomodulation	hiPSCs	Footprint-free MSCs	Wnt3a, Activin A, and BMP4 had synergistic effects on MSC after just 4 days of therapy and microbead encapsulation	Create osteogenesis, chondrogenesis, and adipogenesis lineages without teratoma development in vivo	[139]
		MSCs	Bone tissue	Scaffolds: GO was cross-linked with N-hydroxy succinimide and 1-ethyl-3-(3-dimethylaminopropyl) carbodiimide hydrochloride	Less than 0.5% GO was biocompatible and encouraged osteogenesis and proliferation	[140]
		MSCs	Cardiac tissue	Direct injection of saline 2 × 10^8^ hESC-CMs or 2 × 10^8^ hiPSC-MSCs into the myocardium	No proarrhythmia or tumor formation and improvement of cardiac function	[141]
	Pharmaceuticals	iPSC-derived EVs	Neuron tissue	Electroacupuncture and iPSC-derived extracellular vesicles on mice with ischemic stroke	Treatment for ischemic stroke and damaged tissues	[142]
		iPSC-derived EVs	Neuron tissue	Motor neurons load mRNAs into EVs to control specific processes	Differentiated into motor neurons	[115]
		Cardiac Cells	Cardiac tissue	Post-infarction remodeling, extracellular vesicles released by ISX-9-induced CPCs	Increased angiogenesis, cardiomyocyte proliferation, and used in heart infarction treatment	[143]
Cancer Therapy	Paracrine Effects	Endothelial cells	Breast cancer	Organotypic microfluidic model of human vasculature upregulated secreted factors during cancer cell extravasation	Increased levels of IL-6, IL-8, and MMP-3 and assessment of therapeutic drugs in cancer metastasis	[144]
		iPSC	Cell-derived tumors	Hypoxia-inducible factor-1-alpha-regulated matrix metalloproteinases operate as a mediator downstream of mTORC1	Development of stem cell-derived tumors	[145]
		iPSC	Osteosarcoma	The tumorigenic potential is repressed by suppression of SFRP2, FOXM1, or CYR61	A potential treatment approach is to suppress SFRP2	[146]
	Differentiation	Antigen-specific T cells	Xenograft cancer models	Differentiating CD8ab T cells into antigen-specific TCR	impede the growth of tumors in xenograft cancer models	[147]
		Mouse iPSCs	Tumor vasculature	The recruitment of host endothelium vessels into the tumor is aided by cancer stem cells	Investigate the tumor vasculature and create fresh approaches to targeting	[148]
		Cardiac Cells	Breast Cancer	Without causing cardiomyocyte death, clinically relevant doses of trastuzumab reduced the iPSC-CMs' ability to contract and handle calcium	Mechanism behind the emergence of heart dysfunction is changes in cellular metabolic pathways	[149]
	Biomodulation	NK cells	Ovarian cancer xenograft mode	Comparing T-CAR-expressing iPSC-derived NK cells and non-CAR-expressing cells, CAR exhibit antitumor efficacy	"Off-the-shelf" targeted lymphocytes for immunotherapy against cancer	[18]
		NK cells	Tumor lysis	Enhance cytokines, cytotoxicity against solid and hematologic malignancies, and attracted T cells and anti-PD-1 antibodies	Encouraging the infiltration of T cells to enhance checkpoint inhibitor treatments	[107]
		NK cells	NK cells mediated tumor	Through the expression of CD16A, CD64/16A, and the altered NK cells, tumor cell death was mediated	IgG Fc chimeric proteins and therapeutic mAbs with switchable targeting components	[150]
		Macrophages	Removal cancer cells	CAR expression improves tumor cell phagocytosis, polarizes macrophages, and secretes cytokines	Utilized to eliminate cancer cells	[113]
		Macrophages	Disease models	iPSC line SFCi55-ZsGreen is used to produce terminally differentiated macrophages	Used to track disease model progression in vivo	[151]
		MSCs	in vitro and in vivo Anti-tumor effects	Through apoptotic signaling pathways, TRAIL-iMSCs reduced tumor growth in xenografts of the A549 or MCF-7	High homogeneity therapeutic gene-targeted MSCs for cancer treatment	[152]
		MSCs	Facial Tumor	iMSCs were differentiated by transforming growth factor beta/activin signaling pathway inhibition	Immunomodulatory and anti-inflammatory	[153]
		Cytotoxic T lymphocytes	Cervical cancer	Revealed strong cytotoxicity against cervical cancer after differentiating into HPV16-specific regenerated CTLs	Tumors overwhelm result on epithelial cancers	[154]
Drug Development	Paracrine Effects	Cardiac Cells	Cardiovascular disease	H9C2 cells are protected against stress-induced senescence by blocking the p53-p21 and p16-pRb pathways	Therapeutic approach for cardiovascular disease	[155]
	Differentiation	iPSCs and ESCs	Blood cells	Mature blood cells are formed in part by chromatin state, DNA methylation, and gene expression	the best way to choose iPSCs for clinical purposes	[156]
		iPSCs	Hepatocytes and adipocytes	Transcriptomic and metabolomic effects of the 1p13 rs12740374 variation on cardiometabolic markers	Tools for GWAS variant validation	[157]
		iPSCs	Hematopoietic cells	DNA methylation and gene expression patterns associated with leukemia	Examining the clonal characteristics of human AML	[158]
		iPSCs	Microglia	Exposure to certain stimuli and co-culture with astrocytes to induce microglial differentiation	Similar functional traits of isolated Microglia from the brain	[159]
	Biomodulation	iPSCs	Hematopoietic cells	Myeloid malignancy is caused by the chromosome 7q loss and the splicing factor SRSF2 P95L mutation	Drug discovery and testing are done with hematopoietic cells	[160]
		iPSC-derived MSCs	Immunomodulatory effects	T cell responses as an action of soluble factors and inhibiting the cleavage of caspases	Immunomodulatory effects on T cell responses	[161]
		iPSCs	Strong immunomodulatory	Decreased c-Myc expression and downregulation of the DNA replication pathway	Low oncogenicity and strong immunomodulatory, good potential for therapeutic use	[162]
		Ligament and gingival Cells	iPSC-MSC	To raise the number of Treg cells while decreasing the number of Th1/Th2/Th17 populations and T-cell effectors	Clinical use in therapeutic applications and potent immunosuppressive properties	[163]
		MSCs	Myocardial infarction	Intravenous infusion of 5 × 10^5^ or 1 × 10^6^ hiPSC-CMs	enhance cardiac function in myocardial infarction	[164]
	Pharmaceuticals	Liver cells	Liver diseases	Apolipoprotein B synthesis is inhibited by cardiac glycosides	Treatments for inborn errors of hepatic metabolism	[114]
		Liver cells	Liver fibrosis	qHSC-like cells converted into activated HSCs in culture	Investigate the therapeutic compounds connected to HSCs'	[165]
		Liver cells	Liver injury	Identified key drug transporters and metabolizing enzymes	Utilized in tests for toxicity, excretion, and metabolism	[166]
		Liver cells	Hepatotoxicity assessment	Using confocal and 3D image analysis, several spheroid phenotypes compared multi-parametrically	Differences between the two cell types' pharmacological effects	[167]
		Liver and cardiac cell	Drug efficacy, and toxicity assessment	By the cytochrome P450 enzyme in the liver MPS, cisapride is metabolically converted to nonarrhythmogenic norcisapride	Screening of the liver and heart for medication effectiveness, and toxicity	[168]
		Neuron cells	Neurological mtDNA Disorders	Avanafil drug partially corrects the calcium deficiency in patient NPCs and differentiated neurons	Model for testing drugs for mtDNA diseases	[169]
		Neuron cells	Alzheimer’s Disease	Topiramate is an anti-Ab cocktail comprised of a combination of 27 Ab-lowering screen hits, prioritized hits, and 6 leading compounds	Beneficial in the development of drugs for Alzheimer's disease	[170]
		Cardiac cells	Drug-induced clinical trials	Values of field potential duration prolongation and clinically concentrations were associated	Demonstrate the feasibility of in vitro preclinical studies	[171]
		Cardiac cells	Cardiac disease	Isoproterenol and verapamil 3D-printed an asymmetric, cantilever-based tissue scaffold	Drug discovery via high-throughput screening	[172]
		Macrophages	Modeling of Tissue Resident Macrophage	Growth factors and particular organ specific cues can help macrophages differentiate	iMacs with pro-inflammatory characteristics, mimicking the disease phenotype	[173]
		Cardiac cells	3D-iPSC cardiomyocyte tissues for drug development	iPSC-CM tissues offer blood capillary-like networks and synchronous beating ratios	Compared to 2D-iPSC-CM cells, 3D-iPSC-CM tissues showed hazardous reactions	[174]

### Tissue regeneration

**Fig. 4:**
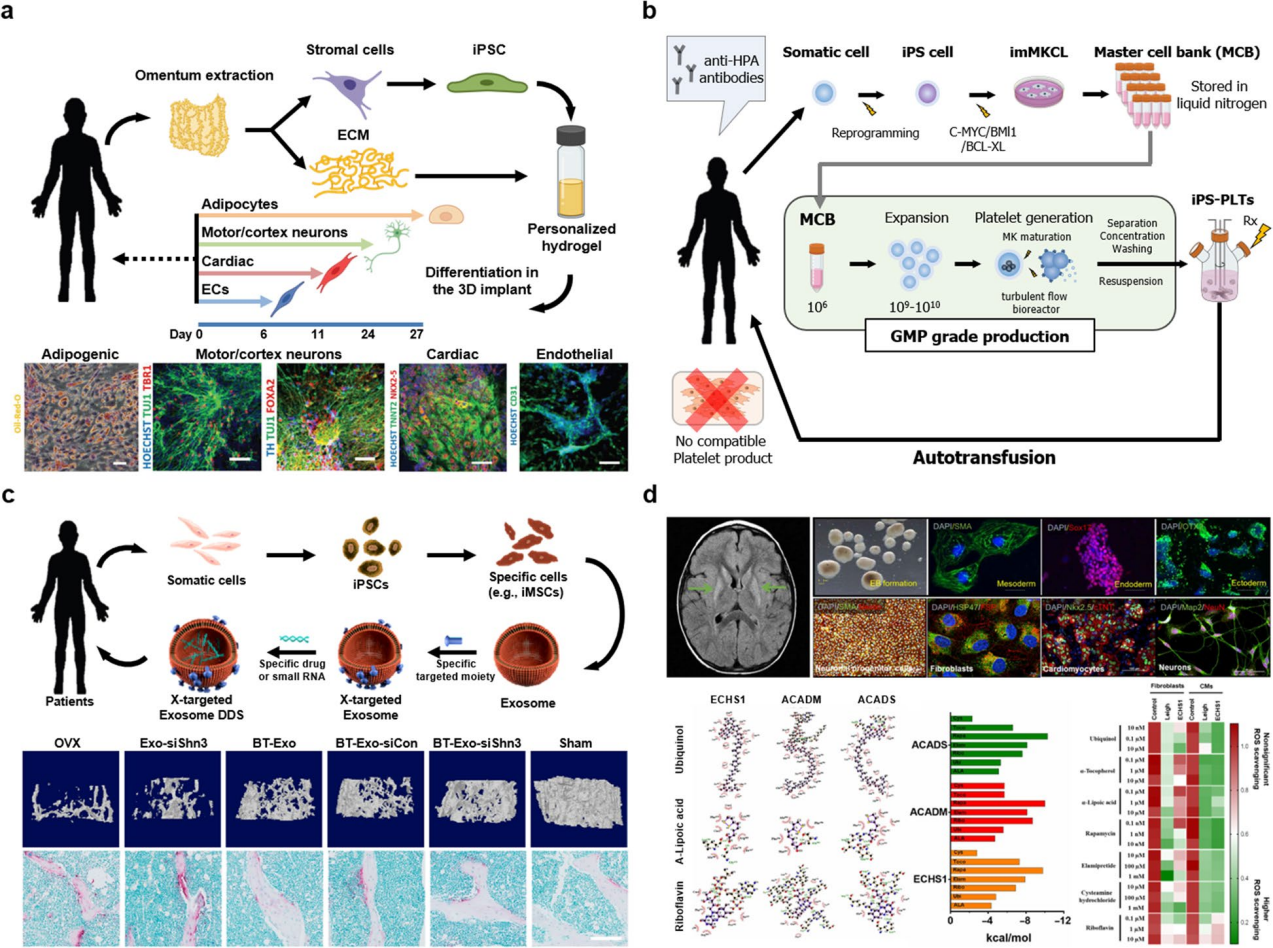
Specialized applications of iPSCs for personalized medicine. **a** Personalized hydrogels for engineering diverse fully autologous tissue implants, which were efficiently generated by combining autologous iPSCs and extracellular matrix. As both the cells and the hydrogels are derived from the patient, they do not induce an immune response. Reproduced with permission from Ref. [175]. **b** The first-in-human clinical trial of iPSC-derived platelets (iPLAT1). The iPLAT1 study completed the administration of iPSC-platelets for the first time and confirmed the safety in an allo-PTR patient who would otherwise have no HPA-compatible donor. No adverse events were observed during the administration of autologous iPLAT1. Reproduced with permission from Ref. [177]. **c** Development of an engineered exosome delivery system. The engineered exosomes, BT-Exo-siShn3, targeted osteoblasts specifically and contained siRNA to silence the Shn3 gene, which enhanced osteogenic differentiation and decreased autologous RANKL expression. Reproduced with permission from Ref. [178]. **d** Drug screening platform using iPSCs derived from a patient with ultrarare diseases. The iPSC platform validated the safety and efficacy of the screened drugs. The efficacy of the screened drugs was also investigated in a patient with Leigh-like syndrome, who showed an enhanced physical state after three years of clinical trials. Reproduced with permission from Ref. [179]

Personalized tissue regeneration can involve either scaffold transplantation or direct injection. For scaffold transplantation, it is important to ensure that the materials, architectures, physicochemical properties, and tissue constructs are individualized according to the patient’s needs. Edri et al. suggested a novel approach for engineering cardiac, cortical, spinal cord, and adipogenic tissue implants from one small tissue biopsy (Fig. 4A). That is, they generated personalized hydrogels by efficiently combining autologous iPSCs and extracellular matrix, where both the cells and the hydrogels are derived from the patient so do not induce an immune response. They suggested promising approach to efficiently bioengineer autologous tissue construct with any tissue type [175]. Moreover, Montgomery et al. introduced a promising strategy to deliver murine iPSC-derived neural progenitors with fibrin-based scaffolds. Owing to their properties suitable for affinity-based drug delivery systems, many studies have been conducted on developing cell-based delivery platform using fibrin scaffolds. They, for the first time, proposed a strategy including a rapid and efficient protocol for forming embryonic bodies from iPSCs and maximizing subsequent neuronal differentiation. They proposed efficient approach for a personalized spinal cord injury therapy [176].

Another approach for tissue regeneration involves directly injecting iPSCs into patients. Immune responses and differentiated states of iPSCs are important issues for this approach. For example, Lu et al. compared the wound healing effect of iPSC-derived therapeutics on non-human primates by subcutaneously injecting autologous and allogeneic iPSCs into immune response-free monkeys. The results demonstrated the superior wound healing capabilities of autologous iPSCs to their allogenic counterparts [90]. Recently, several researchers have reported human clinical trials of iPSCs [13, 180]. Sugimoto et al. proposed the first-in-human clinical trial of autologous iPSC-derived platelets (Fig. 4B), in which iPSCs were efficiently expanded and differentiated during GMP-grade production. The iPSC-derived platelets were then administered to a patient who experienced systemic post-transfusion purpura-like complications and had no compatible donor, with no adverse effects. As the first clinical trial using iPSC-derived platelets, this study present feasibility and significant insight for iPSC-based personalized medicine [177].

### Cancer therapy

The potential applications of iPSCs for personalized cancer treatment can be divided into two. The first application is the replacement or repair of damaged tissue caused by radiotherapy and surgery conducted to eliminate tumors [181]. As autologous iPSCs are free from immune responses and ethical issues, they can provide various strategies to repair damaged tissues by engineering the patient’s cells from healthy tissue. For example, Zhang et al. employed an iPSC-derived conditioned medium to alleviate gamma-irradiation-induced lacrimal gland injury. They found that the iPSC-derived conditioned medium reduced inflammatory responses after radiation therapy by suppressing p38/JNK signaling, which suggests that iPSCs have the potential to treat cancer radiotherapy-related injury [182].

The second application involves the significant advantages of iPSCs for cancer immunotherapies [183]. Although existing cell-based immunotherapies for cancer treatment have undergone substantial advances, limitations such as high cost, difficulty in large-scale production, and unsuitability for lymphopenia patients hinder their widespread clinical use [104]. However, the use of iPSCs combined with engineering strategies can overcome the current limitations of cancer immunotherapy. iPSCs can be continuously expanded and differentiated to acquire an unlimited supply of various immune cells [119]. For example, Iriguchi et al. introduced a scalable method to establish T-cells using iPSCs derived from antigen-specific cytotoxic T-cells or T-cell receptor-transduced iPSCs. They also described culture systems for the efficient differentiation of iPSCs into T-cells. Their study represents a novel strategy for the large-scale production of T-cells and their clinical application to cancer immunotherapy [184]. In addition, the application of CAR engineering to iPSC-derived immune cells can achieve effective treatment by specifically targeting tumor-associated antigens. Li et al. engineered iPSC-derived NK cells to express chimeric antigen receptors (NK-CAR-iPSC-NK cells), which significantly suppressed tumor growth in an ovarian cancer xenograft model while exhibiting reduced cytotoxicity. The proposed NK-CAR-iPSC-NK cells have substantial potential in cancer immunotherapy [18].

### Drug development

Engineering iPSCs is expected to further advance the innovative application of iPSCs to personalized medicine, for example, mutation-specific therapies, early detection strategies, personalized disease prevention, personalized drug testing, and personalized medicine development [183]. In this section, we focus on the personalized application of iPSC-based therapeutics. Several studies have developed nanomedicines from iPSC-derived extracellular vesicles. Extracellular vesicles secreted by iPSCs have great potential for cell-free regenerative medicine [185]. In specific, Cui et. al., engineered an iPSC-derived exosome to develop a bone-targeting gene delivery system (Fig. 4C). The engineered exosomes not only showed intrinsic anti-osteoporosis function but also exhibited an ability to deliver siRNA to osteoblasts to enhance therapeutic effect. Their study shows the potential of exosome for personalized medicine through the development of nanomedicines that can target specific diseases [178]. Besides, various studies have reported the efficacy of iPSC-derived extracellular vesicles in targeting specific diseases such as cardiac diseases [186], ischemic diseases [187], neurodegenerative diseases [188], and cancer therapy [189]. Moreover, with the help of personalized platforms established from iPSCs, it is possible to help patients make rational decisions in clinical trials. Sequiera et al. developed a personalized drug screening platform using iPSCs from a patient with ultrarare diseases (Fig. 4D), then used the platform to evaluate the efficacy of three drugs over three years of clinical trials. The results indicated an enhanced physical state in the patient with Leigh-like syndrome. Moreover, the iPSC-based pre-screening platform helped the patient make safe and effective decisions in a personalized manner [179]. These findings provide next-generation strategies for developing iPSC-based personalized medicine.

## Limitations, challenges, and prospects

### Limitations and challenges

Engineering iPSCs for therapeutic applications has huge potential for personalized medicine, which may be able to overcome the limitations of conventional disease treatments. Despite the many advantages of iPSCs for personalized medicine, there are still several limitations to be a promising tool for therapeutic applications.

First, the reprogramming efficiency, safety and efficacy are major considerations of iPSC-based personalized medicine. The efficiency of iPSC reprogramming is typically low, with the formation of tumorigenesis another drawback to the application of regenerative medicine [190]. Teratoma formation is critical challenge for iPSC-based therapeutic applications [191]. An equally important consideration is the potential for disease development from the viral and non-viral vectors, as well as the reprogramming factors, which may induce a critical condition in the patient [192]. The cell survival rate after transplantation is worthy of consideration. The number of transplanted cells engrafted in the damaged tissues depends on the disease condition and age of the patient. Additionally, once iPSCs are familiarized with the specific treatment region, they are generally targeted by innate and adaptive immune responses via the host body’s immune system [193]. Recently, various studies have been proposed to reduce the immune rejection of iPSCs using CRISPR/Cas9-mediated genetic engineering [194].

Second, the lack of internationally approved regulatory guidelines for the production protocols of in-hospital iPSCs, commercialized iPSCs, and personalized iPSC lines hinders the application of iPSCs to personalized medicine. Moreover, in-process sterility systems for checking bacterial contamination (*Mycoplasma*) or viral contamination should be established according to the recommended quality control guidelines of USP, European Pharmacopoeia, or other recognized regulatory bodies. In addition, internationally accredited and standardized methodologies for delivering iPSCs to the targeted area have not yet been established. This is currently the greatest limitation, especially for critical organs, as well as ensuring reliable clinical staff for the iPSC delivery program. To resolve abovementioned limitations, the global alliance for iPSC Therapies (GAiT) has recently published the minimum requirements of quality control testing of iPSC [195].

Finally, regarding the production of therapeutic iPSCs, high costs associated with their production, characterization, and quality assessment are also a major limitation of therapeutic applications and commercialization [192]. First, iPSC identification should be performed during treatment, from the biopsy stage to the end-product stage, by single tandem repeat profiles in an accredited laboratory to confirm and ensure cell activities. Second, purity validation must also be confirmed for patient safety. To confirm patient wellbeing, internationally recognized and standardized purity qualification methods should be established from the sample collection stage to the end stage of therapeutic application. Third, constant reproducibility should be maintained from the initial stage of the iPSC production line through to the end point of personalized treatment, which requires consideration of the isolation methods, cell culture conditions, engineering strategies, and methods of application [192]. Finally, after the production of commercialized therapeutic iPSCs, maintaining the appropriate conditions in storage and transportation facilities, e.g. pH, temperature, and humidity, represents an immense challenge [196]. These issues can be overcome with help of fully automated production systems. Recently, Paull et al., developed a modular, robotic platform for automated iPSC reprogramming, characterization, and differentiation to achieve minimal manual intervention [64]. Although some automated isolation, reprogramming, expansion, and in-process quality checking systems have been established, these should be integrated into an organized system [65].

### Prospects

Undoubtedly, iPSC-based regenerative therapy will become an important aspect of personalized medicine in future, with abundant research already bringing us closer to this goal. Notably, the development of iPSC lines has eliminated the ethical issues and religious concerns associated with embryonic stem cells but maintained their excellent pluripotency properties [190]. The risk of immune rejection has already been reduced and will likely be completely removed in the near future. Additionally, with the development of personalized iPSC line banking, it is now possible to store patient-specific genetic and immunological information and apply personalized regenerative therapy via automated procedures according to GMP regulatory criteria [86]. Some fully automated iPSC line production techniques with integrated quality assurance have also been proposed [87]. Although establishing personalized iPSC line bank is costly and time consuming, it would be a reliable and effective solution for personalized therapy in the future. Additionally, regarding disease modeling, disease-causing factors can now be identified by the microfluidics model for different patients. High-throughput screening for drug testing and toxicity prediction is also undergoing continuous development. In the meantime, gene editing technology to correct mutations for genetic disease treatment has been made possible through CRISPR, TALEN, and ZINC finger techniques [55]. Nevertheless, the mass production of commercialized therapeutic iPSCs according to the proper regulatory guidelines, involving appropriate quality assessments, and conducted in accredited GMP-compliant facilities remains a substantial challenge for regenerative therapy. To accomplish this, the Global Alliance for iPSC Therapies has proposed critical quality attributes and recommended test methods for producing clinical-grade iPSC lines for therapeutic applications [195]. Once the various limitations and challenges are overcome, engineered iPSCs could become a key tool for the personalized medical treatment of many life-threatening diseases.

## Conclusion

Personalized medicine provides a tailored medical treatment based on the unique clinical, genetic, and environmental characteristics of individual patients. Moreover, engineering strategies offer a wide range of opportunities for advancing iPSC-based personalized medicine. In this review, we summarize how engineering strategies have been applied to advance iPSC-based personalized medicine by categorizing the process into three distinctive steps: 1) production of therapeutic iPSCs; 2) engineering of therapeutic iPSCs; and 3) application of engineered iPSCs. For each step, we discuss the various engineering approaches and their implications. Although there are still many limitations to the use of iPSCs in personalized medicine, including reprogramming efficiency, large-scale production of therapeutic iPSCs, the possibility of teratoma formation, commercialization, and regulatory approval, the engineering strategies presented in this review can help overcome these limitations. Undoubtedly, iPSC-based personalized therapy will become a valuable and innovative medical solution in the near future.

## Data Availability

Data will be made available on request.

## Abbreviations

iPSCs: Induced pluripotent stem cells
OCT3/4: Octamer-binding transcription factor 3/4
SOX2: Sex determining region Y-box transcription factor 2
c-MYC: Cellular MYC
KLF4: Kruppel-like factor 4
GMP: Good manufacturing practice
GATA4: GATA Binding Protein 4
POU5F1: Octamer-binding transcription factor 4
LIN28: Cell lineage abnormal 28
TRNT1: TRNA-nucleotidyltransferase 1
EGFP: Enhanced green fluorescent protein
GBX2: Gastrulation brain homeobox 2
SP8: Specificity protein 8
ZIC1: Zinc finger protein of cerebellum 1
CRISPR/Cas9: Clustered regularly interspaced short palindromic repeats-associated protein 9
FDA: Food and Drug Administration
ISO: International Organization for Standardization
CAR-T-cell: Chimeric antigen receptor T-cell
NK cell: Natural killer cell
BMP: Bone morphogenetic protein
FGF2: Fibroblast growth factor 2
VEGF: Vascular endothelial growth factor
HGF: Hepatocyte growth factor
EGF: Epidermal growth factor
PDGF-BB: Platelet-derived growth factor-BB
TFGβ: Transforming growth factor beta
HPA: Human platelet antigen
AD: Alzheimer's disease
TALEN: Transcription activator-like effector nucleases
